# Bacterial Community Structure Responds to Soil Management in the Rhizosphere of Vine Grape Vineyards

**DOI:** 10.3390/biology13040254

**Published:** 2024-04-12

**Authors:** Barnabás Kovács, Marco Andreolli, Silvia Lampis, Borbála Biró, Zsolt Kotroczó

**Affiliations:** 1 Institute of Viticulture and Enology, Hungarian University of Agriculture and Life Sciences, 8360 Keszthely, Hungary; 2 Department of Biotechnology & Verona University Culture Collection-Department of Biotechnology (VUCC-DBT), University of Verona, 37134 Verona, Italy; marco.andreolli@univr.it (M.A.); silvia.lampis@univr.it (S.L.); 3 Department of Agro-Environmental Studies, Hungarian University of Agriculture and Life Sciences, 1118 Budapest, Hungary; biro.borbala@gmail.com (B.B.); kotroczo.zsolt@gmail.com (Z.K.)

**Keywords:** vine grape rhizosphere, cultivation method, DGGE, bacterial diversity, sustainable viticulture

## Abstract

**Simple Summary:**

Viticulture is one of the most resource-demanding agricultural sectors worldwide. Therefore, if we would like to find a way to make it more sustainable, we need to understand how cultivation methods affect the interactions between vine grape and its environment. However, very little is known about what bacteria live in the root system of grapes. Furthermore, we have even less knowledge about how the microorganisms’ composition might be changed due to various tillage methods and soil disturbances. This study examined and compared the soil–bacterial microbial composition of three experimental plots using laboratory techniques and up-to-date molecular methods. We investigated soil microbial communities’ composition and some cultivation practices’ effects. Our results show that intensive tillage significantly and negatively affected soil bacterial community structure and diversity.

**Abstract:**

The microbial communities of the rhizospheres of vineyards have been subject to a considerable body of research, but it is still unclear how the applied soil cultivation methods are able to change the structure, composition, and level of diversity of their communities. Rhizosphere samples were collected from three neighbouring vineyards with the same time of planting and planting material (rootstock: Teleki 5C; *Vitis vinifera*: Müller Thurgau). Our objective was to examine the diversity occurring in bacterial community structures in vineyards that differ only in the methods of tillage procedure applied, namely intensive (INT), extensive (EXT), and abandoned (AB). For that we took samples from two depths (10–30 cm (shallow = S) and 30–50 cm (deep = D) of the grape rhizosphere in each vineyard and the laboratory and immediately prepared the slices of the roots for DNA-based analysis of the bacterial communities. Bacterial community structure was assessed by means of PCR-DGGE analysis carried out on the v3 region of 16S rRNA gene. Based on the band composition of the DGGE profiles thus obtained, the diversity of the microbial communities was evaluated and determined by the Shannon–Weaver index (H′). Between the AB and EXT vineyards at the S depth, the similarity of the community structure was 55%; however, the similarity of the D samples was more than 80%, while the difference between the INT samples and the other two was also higher than 80%. Based on our results, we can conclude that intensive cultivation strongly affects the structure and diversity of the bacterial community.

## 1. Introduction

One of the key features of sustainable agriculture is that it is diverse, resilient, and adaptable. Therefore, the amount and diversity of microbes in soils are significant, as they play a crucial role in the uptake and recycling of soil nutrients and formation [1]. The microbiological communities of the rhizospheres of vineyards have been subject to a considerable body of research [2]. However, it is still unclear how the applied soil cultivation methods change their microbial communities’ structure, composition, and level of diversity [3,4].

Anthropogenic impacts and changing climatic conditions cause biotic and abiotic stresses and modify soil bacterial and plant diversity. Fertilizer use, farming, and tillage practices in agroecosystems are causing changes in the soil microbiome [5].

In addition to fungi, soil bacteria are essential partners in the rhizosphere for plants such as vine grapes. Soil bacteria can stimulate plant growth by increasing the availability of nutrients such as N, P, and Fe, producing phytohormones, limiting plant pathogenic organisms’ activity [6,7,8], and indirectly affecting soil fertility and health [9,10]. The beneficial rhizosphere bacteria are called Plant Growth Promoting Rhizobacteria (PGPR) based on their plant-helping effects. According to our current knowledge, these can make up 2–5 percent of the rhizosphere bacterial community; their presence primarily depends on the plant, but abiotic conditions also exhibit a strong influence [11]. The vine grape rhizosphere contains many diverse bacteria that interact with different plant parts. These bacteria can also be transferred to wine, affecting its quality [12]. Molecular biology studies have reported that the microbiome of vine grapes is related to the location of the vineyard and environmental factors [13]. Studies using conventional and molecular biology methods have shown in Merlot that the soil microflora influences the microbiota of the grapevine plant. This affects the epiphytic bacterial communities of grape berries, leaves, buds, and soil [14].

The diversity of bacteria in the rhizosphere of a grapevine plant is essential for healthy plant development. The role of rhizosphere bacteria is highly diverse and complex within the soil–microbe–plant system. In many cases, plant diseases are suppressed by various microbes that inhibit plant pathogens by producing an enzyme (chitinase) that limits the growth of phytopathogenic fungi [15,16].

The vulnerability of vineyards through their root systems receives little attention from both practitioners and scientists from a plant health perspective. Therefore, the community’s presence and proportion of below-ground pathogenic microbes is poorly understood [3]. However, some agrotechnical operations involving mechanical soil disturbance and root cutting may pose a significant risk of penetration by providing surfaces for inoculation by pathogens [17,18]. Among the pathogenic organisms, fungi and nematodes are the ones that act as significant plant pests in the rhizosphere. Pathogenic bacteria can only penetrate and damage the plant through injuries [19]. Following this recognition, most research has focused on the composition of the microbial communities [14], the proportion of pathogenic strains [15], and, most importantly, prevention. This has facilitated the spread of control practices such as pre-planting soil disinfection, the use of more resistant varieties [16], and the deposition of dead plant parts instead of rotation [17,18].

Soil cultivation practices in plantations are constantly developing to meet new requirements. Climate change, the acquisition of new machinery related to technological progress, and continually changing consumer demands (e.g., the need for environmental sustainability) represent new challenges for farmers [19,20]. The soil conditions of plantations are also influenced by the cultivation methods used [21,22]. As mentioned above, this, together with the challenges posed by climate change, uneven rainfall patterns, and extreme weather conditions, must be considered by farmers. Efforts should, therefore, be made to create conditions that provide the right ecological conditions for both the cultivated crop and the soil organisms. Soil biodiversity is, thus, key to this aspect [23], and the most anthropogenic and unfavorable land-use practices must be replaced by an approach that exploits the activity of microorganisms well adapted to local conditions. It is therefore essential to detect soil microbes and understand their role in soil processes.

Several studies confirm that intensive tillage practices cause significant changes in soil microbial composition [2,14,24], with edaphon diversity decreasing in intensively cultivated plantations [24,25], similar to suppressive values. Based on the findings of Huber et al. [3] and Alabouvette [26], the lower levels of suppressive appear as a side effect of intensive soil cultivation where the soil of the plantation resistance against phytopathogenic organisms decreases, and this is often associated with lower organic C intake.

Based on the available literature, we aspired to determine whether the intensity of the cultivation methods could change the level of diversity of bacterial communities and, consequently, the proportion of pathogenic strains in the grape rhizosphere. The relevance of our question is underlined by its potential benefits since the indirect impact of reducing soil tillage intensity might result in lesser exposure of the plant to pathogens underground. Alongside the expected increase in diversity, there would also be the ecological advantage of reducing the intensity of soil agitation.

## 2. Materials and Methods

### 2.1. Sampled Vineyards

**Figure 1:**
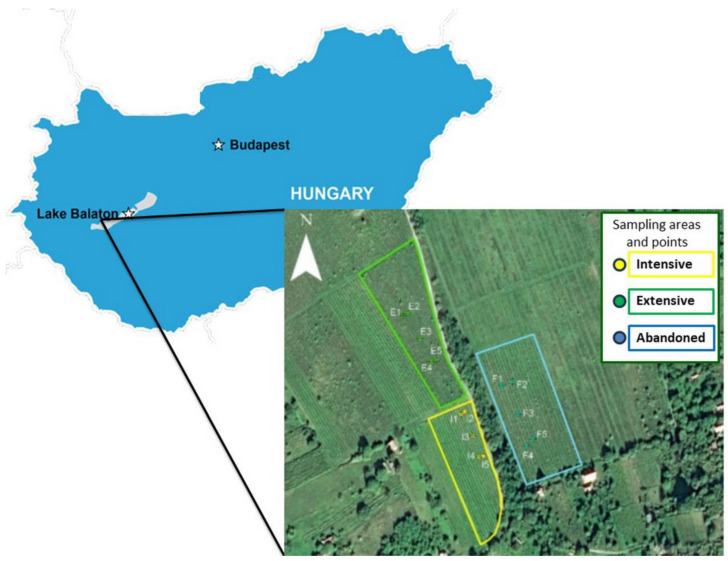
The location of the plantations on Saint George Hill, Hungary.

We used three vineyards in our examinations in Badacsony, Hungary’s wine region, on slight slopes at the northern foot of the volcanic Saint George Hill (Figure 1). The sampling areas are right next to each other, making them direct neighbors (extensively cultivated vineyard: 46°85′304″ 17.44237N 17°44′237″ E; intensively cultivated vineyard: 46°85′218″ N 17°44′293″E; abandoned vineyard: 46°85′268″ N 17°44′351″ E).

All three plantations were planted by the same company at the beginning of the 1980s, with grafts on T.5C (Berlandieri × Riparia) rootstock and Müller Thurgau scion (*Vitis vinifera* L.) with a low number of plants (less than 2500 plants per hectare). The cultivation methods used on the plantations for the last 15 years have also been different.

In 2018 (timing of the examination was 25 April), disking, which means a disturbance within the top 20 cm of the soil, was applied once at the beginning of April as soil tillage on the intensely cultivated plantation (INT). In the extensively cultivated vineyard (EXT), no procedure was applied that would disturb the soil. Before our spring sampling, 12 months had passed with no mechanical soil tillage, which was the appliance of a disk harrow 0–20 cm deep. Instead of soil-disturbing weeding processes, grass cutting was performed two times during the vegetative period. The single curtain training system allowed the agronomist to let the grass grow higher without risking the humidity becoming higher and the cluster zone becoming exposed to pathogen fungi.

For the third vineyard, the abandoned field (AB), no procedure had been applied in the past 17 years that would disturb the soil, though grass cutting had been conducted yearly, specifically once in the vegetative period.

### 2.2. Sampling

Sampling took place in spring at the end of April. Five samples were taken from each vineyard using a manual stainless steel soil sampler (similar configuration to a gouger auger) at a distance of 25 cm from the trunk at a depth of 10–30 (S = shallow) or 30–50 cm (D = deep) to examine bacteria communities of the rhizosphere of vine grape, with samples also taken at the depths of 0–30 and 30–60 cm for agrochemical examination.

For laboratory analysis, the samples were homogenized per vineyard from the same depth (from 5 samples, 1 composite sample (approximately 250 g) was obtained to represent the studied field) and were placed in sealed plastic bags for microbial community assessment. The composite samples were brought to the lab in a freezer bag and stored in the freezer at −20 °C until microbiological examination [21]. The same steps were taken for agrochemical examination until transportation was completed, and the samples were then brought to an accredited soil laboratory for analysis.

### 2.3. Physical Parameters

The analysis of physical parameters for the soil tests was carried out by the accredited laboratory of the NAIK-Research Institute for Viticulture and Oenology, Badacsony, Hungary. During the tests, the pH values and conductivity of the soil samples were determined with a Consort C830 device (Cole-Parmer, St Neots, Cambridgeshire, UK), and element content was analyzed with a GBC 932 Plus Atomic Absorption Spectrophotometer (GBC Scientific Equipment Ltd., Dandenong, Australia). The N-forms of the samples were determined using TECATOR BD20 shredders (Foss Kjeldahl, Apeldoorn, The Netherlands) and Kjeltec 2200 automatic distillation devices (LabMakelaar, Zevenhuizen, The Netherlands), while a Scheibler calcimeter was used to determine the amount of CaCO_3_. The total content of organic matter was analyzed using the Hoogsteen method [22].

### 2.4. Soil DNA Extraction

From each frozen (−20 °C) soil sample, rootlets were taken (7–8 cm) and sliced randomly to about ~1 mm long parts using a sterile lance. The DNA from the subsamples (0.25 g of field-moist soil from the rhizosphere and from each rootlet) was extracted using the FastDNA^®^ Spin Kit for soil (MP Biomedicals, LLC, Illkirch-Graffenstaden, France) following the manufacturer’s protocol. DNA extracted from soil samples was checked for quality on 1% *w*/*w* agarose gel via electrophoresis at 100 V for 20 min.

### 2.5. PCR Amplification of 16S rRNA Gene

Eubacterial 16S rRNA fragments were amplified using fD1 and rP2 primer as described by Weisburg et al. [27]. The further nested PCR was performed by amplifying the hypervariable V3 region with p2 and p3 primers as reported in Muyzer et al. [28]. PCR products were run on agarose gel in Tris–borate–EDTA buffer for 15 min at 100 V to check the quality of amplicons obtained.

### 2.6. Denaturing Gradient Gel Electrophoresis (DGGE)

DGGE was performed with a DcodeTM Universal Mutation Detection System (Bio-Rad Laboratories Inc., Hercules, CA, USA). PCR products were loaded into 8% polyacrylamide gel with a 30% to 60% denaturant gradient (where 100% denaturant contains 7 M urea and 40% *v*/*v* formamide) and run as previously described [29].

The gels were thus stained for 30 min through the use of Eurosafe (EuroClone SpA, Milan, Italy). The similarity among the different DGGE profiles was evaluated by UPGMA clusters, and the resulting dendrogram was visualized through the UVI bandmap software package V11.11 (UVI tec Ltd., Cambridge, UK) [30].

### 2.7. Sequence Analysis of DGGE Bands

The most representative bands were excised with a sterile blade and transferred to 30 μL TE (Tris-EDTA buffer, Sigma-Aldrich, St. Louis, MO, USA). DNA was allowed to diffuse into the TE by incubation at 37 °C for 3 h. A total of 3 μL of the DNA solution was used as a template and re-amplified using non-GC clamped primers p1 and p2 [28]. PCR products were transferred in *Escherichia coli* Xl1-blue using the Promega pGEM-T vector system (Promega, Madison, WI, USA) according to the manufacturer’s instructions, and the insert was further sequenced by Primm Srl (Milan, Italy). The obtained sequences were used as BLAST queries [31] against the GenBank (NCBI) database [32].

## 3. Results and Discussions

### 3.1. Results of Soil Analysis

It is necessary to evaluate the agrochemical results in light of the fact that no nutrient replenishment was carried out in the case of any of the plantations, so the origin of any differences must be sought in the different levels of load resulting from the different number of plants in the vineyards due to a shortage of grapevines due to pathogens (Table 1). The levels of sulfur (S) and copper (Cu) are exceptions because the level of these elements grows with more intense plant protection activities (concentration and density of spraying).

At both sampling depths, the three plantations have weakly acidic, weakly calcareous, and low-salt soils.

According to the laboratory soil analyses’ evaluated outcomes [36], the soils were slightly different regarding their physical and agrochemical characteristics. The difference was in the amount of organic matter; in the INT plantation it was lower at both sampling depths than in the other two plantations. The level of Cu (Table 1) was higher in the EXT plantation and at the deeper samples in the AB plantation too compared to the INT plantation, and this difference is possibly explained by the applied cultivation methods. The EXT vineyard is managed by certified organic methods, which means only copper (Cu)-based plant protection can be applied against the most damaging above-ground fungi, such as downy mildew. Also, in the case of the AB plantation a decade ago, copper was the most often used pesticide, and in the last non-cultivated period it moved down to deeper zones. Although copper content is not particularly high compared to the average of European vineyards [37], it certainly had an impact on both the fungal [38] and bacterial communities [39]. Soil-dwelling fungi are more sensitive to copper than bacteria [40], so it is necessary to account for this effect at plantations with higher values. However, the pH value and disturbance significantly affect the bacterial community [41] and the diversity values [42].

According to the criteria system of [41], the layer of soil deeper than 30 cm is immune to *Phylloxera* (over 85% silicate content, total ratio of humus is lower than 1%). Regarding the consistency of the soil, the intensively cultivated area was the least compacted (still it was compacted based on the average of the measured (n = 10) values: 3.9 MPa/19 mm), while on the other hand the EXT plantation exceeded it by 5% and the AB plantation by 22.5%.

### 3.2. PCR-DGGE on the Grapevine Rhizosphere among Different Vineyards

**Figure 2:**
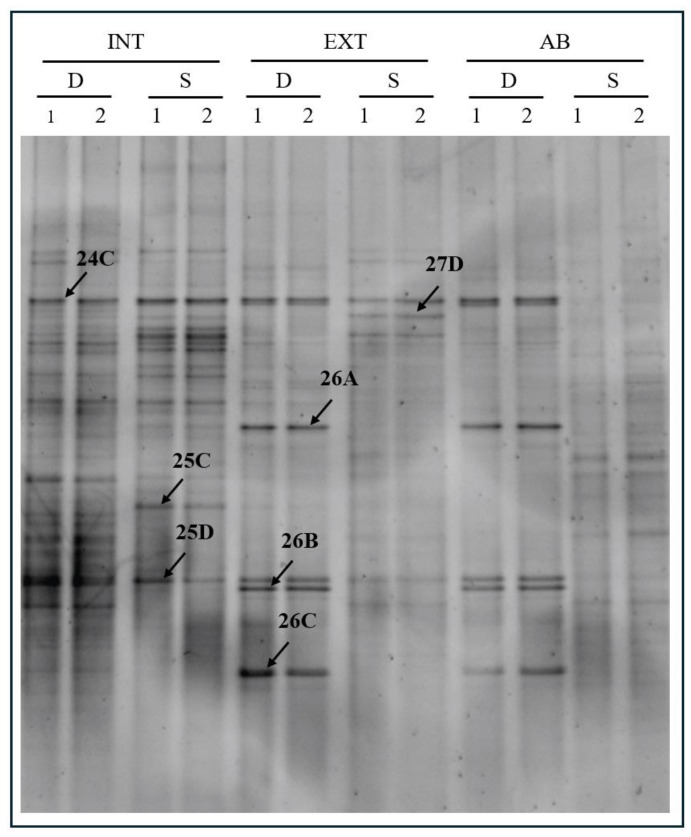
PCR-DGGE analysis performed in duplicated (1 and 2) on rhizosphere soil samples collected from plants exposed to intensive (INT), extensive (EXT), and abandoned (AB) tillage methods at two different depths: shallow (S) and deep (D). Arrows indicate bands excised from the gel for sequencing.

PCR-DGGE analysis was performed to compare the grapevine rhizosphere microbial communities grown with three different tillage methods: intensive (INT), extensive (EXT), and abandoned (AB). Moreover, two distinct depths (S and D) were investigated. The results evidenced the presence of several bands in the DGGE profiles obtained from different samples, indicating that abundant and various bacterial communities are present (Figure 2).

**Figure 3:**
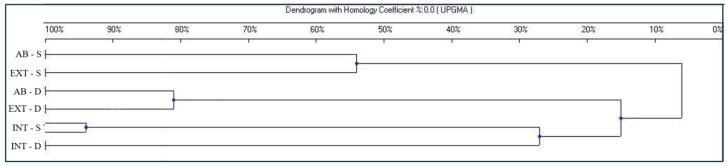
Dendrogram indicating the similarity indices of the different DGGE profiles based on samples collected from intensive (INT), extensive (EXT), and abandoned (AB) tillage methods at two different depths: shallow (S) and deep (D).

The further statistical analyses (Figure 3) evidenced a single cluster harboring both INT samples with only 20% similarity to EXT and AB samples, corresponding to what was previously expected based on the literature [43,44]. Thus, in this work, the intensity of cultivation resulted in the most significant variable, which differentiated the community structure of soil bacteria through pesticides significantly affecting the microbiological population in the grapevine rhizosphere [45].

Moreover, the dendrogram evidenced two more clusters containing the D and S samples, respectively. Therefore, the sampling depths were the main cause of bacterial speciation among abandoned and extensive tilling. The higher similarity percentage between AB and EXT samples compared to INT samples suggests that the main effect on these two vineyards may be the chopper accumulated in the soil over decades. The constant occurrence of this accumulating heavy metal in the measured depths of the soil may have a stronger impact on the bacterial community than the differences between the current plant protection activities in the vineyards.

### 3.3. Identification Analysis of the Most Representative DGGE Bands

Seven bands were excised and sequenced (Table 2) that were retrieved from INT and EXT DGGE profiles.

Band 25C showed high similarity to *Nordella oligomobilis*, a species that belongs to the *Rhizobiales* order. *Rhizobiales* bacteria are often found in root samples with high abundance, presumably because of the active preference of plants driven by chemoattraction via secreted photoassimilates [11]. The only occurrence of it for the INT-S sample—according to the data of Table 1—does not relate to the lower nitrogen content, which could be an explanation.

Band 25D corresponding to Pseudomonas genus, *Gammaproteobacteria*, in our samples is described as rhizospheric PGP rhizobacteria. Additionally, aflatoxin degradation capability and an antagonistic effect against pathogens were detected. Their absence from INT-D samples may be caused by the intensive vertical disturbance of the soil, resulting in a less favorable community structure for the taxa. However, more intensive cultivation can generally be favorable for the total number of bacterial species in the bacterial community [51]. It should be noted that the sequencing analysis also detected the same phenomena.

The *Thiophaeococcus* genus was found in the INT-S samples. It belongs to the class *Gammaproteobacteria*, order *Chromatiales*. *Chromatiales* typically grow under anoxic conditions in the light using sulfide as a photosynthetic electron donor, which is oxidized to produce sulfate. The occurrence of the representatives from the most disturbed sample is unexpected due to the air-supplying effect of soil cultivation in the shallow soil zones. However, the second highest level of sulfate (Table 1) in the INT after EXT vineyards may explain why the environment was favorable for the representatives of this class, though it would still be more expected in the deeper zone. A temporary wet and anaerobic period after intense rainfall could complete the explanation, but precise meteorological data are unavailable.

However, our methodology of taking the rhizosphere samples allowed us to divide (references listed in the right column of Table 2) bulk soil and rhizosphere from the seven most abundant strains studied, only three of which are rhizosphere-specific (Table 2). Validov et al. (2007) [42] found in their study that samples from bulk soil have *Rhizobiales* (*Alphaproteobacteria*) and *Xanthomonadales* (*Gammaproteobacteria*) in lower abundance than vine grape root samples. Based on this reference, four of the seven taxa resulted from the sequencing of DGGE bands often occur in vine grape root and rhizosphere soil samples. According to the findings of Klaupfel (1993) [61], those bacteria growing farther from the root surface or growing in nutritionally poorer root segments will not contribute significantly to the total population. It follows that the most abundant species we detected are the ones from the part of the root or rhizosphere where the cooperation between the plant and microbes is the most intense.

## 4. Conclusions

Based on the results of our DNA-based molecular diagnostic tests, it can be concluded that, as expected, the EXT and AB plantations show a significant similarity in the composition of the bacterial community. The more than 80 percent match in the DNA pool extracted from the deeper layer (30–50 cm) allows us to conclude that tillage, which disturbs the surface only rarely, and even then horizontally, does not significantly change the properties of the soil either from an abiotic or biotic point of view, thus not indirectly affecting the composition of the bacterial community.

From the agrochemical data, it emerges that the accumulated copper content in this layer (due to the cultivation practices of previous decades and, in the case of the extensive plantation, the current practice) must be considered as a toxic heavy metal. However, at the same depths for the two areas, the amount of copper is almost the same, and we can see a difference in the intensive area compared to the other two in this respect as well.

The similarity in the community pattern between the two areas shows that the abandonment of soil disturbance, plant protection, and soil compaction for more than a decade in areas with such characteristics does not result in the development of greater diversity in the soil bacterial community compared to extensive cultivation. Presumably, a drastic change appears after the abandonment of mowing with the cessation of monoculture, reaching the next phase of the succession with the appearance of shrubs and trees.

Intensive treatment is separate, with less than 20% being matched with the other two. The vertical disturbance is associated with the destruction of obligate aerobic and anaerobic organisms and, thus, community realignment. In the same way, the presence of dead plant organic matter introduced by rotation also increases the ratio of saprophytes and opportunistic pathogenic species in layers further from the soil surface. Intensive disturbance in such structureless sandy soils also prevents soil moisture conservation and SOC formation. As a result, the edaphon, the community of microbes found in the rhizosphere and its environment, can be exposed to other stress effects, such as water and nitrogen sources that are more difficult to access, which can be an inhibiting factor that modifies the community’s composition.

## Figures and Tables

**Table 1:** Soil parameters, nutrient content, and the evaluated agrochemical results for the experimental vineyards at ‘Saint George Hill’ [33,34,35].

Sample Labels:	INT-S (0–30 cm)	INT-D (30–60 cm)	EXT-S (0–30 cm)	EXT-D (30–60 cm)	AB-S (0–30 cm)	AB-D (30–60 cm)
pH (H_2_O)	7.48	7.74	6.89	6.93	6.16	6.35
pH (KCl)	7.09	7.36	6.55	6.60	5.41	5.70
	slightly acidic	slightly acidic	slightly acidic	slightly acidic	slightly acidic	slightly acidic
KA (soil texture)	23	23	23	25	26	25
	coarse sand	coarse sand	coarse sand	sand	sand	sand
Total salt (m/m%)	<0.02	<0.02	<0.02	<0.02	<0.02	<0.02
	low salinity	low salinity	low salinity	low salinity	low salinity	low salinity
CaCO_3_ (m/m%)	0.17	0.25	0.13	0.35	0.22	0.30
	slightly calcareous	slightly calcareous	slightly calcareous	slightly calcareous	slightly calcareous	slightly calcareous
Organic matter (SOC) (m/m%)	0.63	0.58	0.75	0.71	0.78	0.89
	poor	poor	medium	medium	medium	medium
P_2_O_5_ (mg/kg)	278.0	296.0	228.6	234.2	126.69	202.93
	good	very good	very good	very good	good	very good
K_2_O (mg/kg)	88.4	66.0	240.35	360.10	140.20	188.45
	very good	very good	very good	very good	good	very good
(NO_3_ + NO_2_)-N (mg/kg)	3.79	4.33	3.20	2.40	2.25	2.31
	low	low	low	low	low	low
Na (mg/kg)	6.45	11.9	11.30	2.10	1.08	21.10
	optimal	optimal	optimal	optimal	optimal	optimal
Mg (mg/kg)	43.1	43.6	56.5	46.7	42.0	33.1
	medium	medium	medium	medium	medium	poor
SO_4_-S (mg/kg)	36.8	40.7	46.29	41.77	30.25	21.33
	good	good	good	good	good	good
Mn (mg/kg)	109	99.1	82.92	75.10	55.70	60.8
	sufficient	sufficient	sufficient	sufficient	sufficient	sufficient
Zn (mg/kg)	6.05	5.84	8.65	6.56	4.21	3.95
	good	good	good	good	good	good
Cu (mg/kg)	69	37.9	52.28	42.48	28.60	47.92
	sufficient	sufficient	sufficient	sufficient	sufficient	sufficient

**Table 2:** Sequencing analysis of bands excised from DGGE profiles.

Band	Closest Bacterial Strain	Accession Number	Class	Occurrence in Samples	Occurrence According to the Literature
	(Identity %)				
24C	*Flavobacterium* sp. HS916 (100)	NR_165696	Flavobacteriia	All	Polluted bulk soil [46]
25C	*Nordella oligomobilis N21* (100)	NR_114615	Alphaproteobacteria	INT-S	Environmental uncultured (order: Rhizobiales) bacteria [47]
25D	*Pseudomonas qingdaonensis JJ3* (100)	NR_169411	Gammaproteobacteria	All	Aerobic bacteria from peanut rhizosphere with aflatoxin degradation capability [48], head rot of lesions of broccoli (*Brassica oleracea* L. var. *italica* Plenck) [49], PGPR bacteria in Zea mays’s rhizosphere with resistance of heavy metals (e.g., Cu) [50]
	*Pseudomonas flavescens* NBRC 103044 (100)	NR_114195	Gammaproteobacteria	All	Rhizosphere of tomato, soil, ater [42]
26A	*Arthrobacter ginkgonis SYP-A7299* (99.43)	NR_156061	Actinobacteria	AB-D, EXT-D, INT-S, INT-D	Aerobic strain from the rhizosphere of *Ginkgo biloba* [51]
	*Galactobacter valiniphilus JZ*7 (99.43)	NR_165018	Actinobacteria	AB-D, EXT-D, INT-S	Raw cow milk sample [52]
26B	*Flavobacterium arsenitoxidans* S2-3H	NR_134726	Flavobacteriia	AB-D, EXT-D	Arsenate-oxidizing bacterium from contaminated bulk soil [53]
	*Flavobacterium eburneum* SA31 (98.94)	NR_156035	Flavobacteriia	AB-D, EXT-D	Soil (aerobe) [54]
26C	*Hyphomicrobium vulgare* JCM 6889 (98.83)	NR_104697	Alphaproteobacteria	AB-D, EXT-D	Sea water, river [55]
	*Hyphomicrobium aestuarii* ATCC 27483 (98.83)	NR_104954	Alphaproteobacteria	AB-D, EXT-D, INT-D	River [56]
27D	*Thiophaeococcus mangrovi* JA304 (93.4)	NR_042643	Gammaproteobacteria		Mud of brackish water [57], obligate anaerobe [58]
	*Thiohalocapsa halophila strain* DSM 6210 (93.4)	NR_115076	Gammaproteobacteria	EXT-S, INT-S, INT-D	Anoxic sediment in a marine aquaculture pond, phototrophic [59] it has a requirement for NaCl [60]

## Data Availability

Data are contained within the article.
